# Response to operative experience in paediatric orthopaedics in UK trainees achieving a Certificate of Completion of Training in trauma and orthopaedic surgery: a descriptive analysis of national e-logbook data

**DOI:** 10.1308/rcsann.2025.0085

**Published:** 2026-01-20

**Authors:** D Bose, G Pattison

**Affiliations:** ^1^ University Hospitals Birmingham NHS Foundation Trust, UK; ^2^ University Hospitals Coventry and Warwickshire NHS Trust, UK

We read with great interest the paper by Tom Barrow and his co-authors regarding operative experience in paediatric orthopaedics in UK trainees.^1^

The Specialist Advisory Committee (SAC) in Trauma and Orthopaedics advises the Joint Committee on Surgical Training of the four UK and Ireland royal surgical colleges. Each training programme has an SAC liaison member who advises on the high-stakes training decisions, including the awarding of a Certificate of Completion of Training (CCT). The General Medical Council reviews all surgical curricula every 3 years and commissions the SAC to advise on any changes. SAC members have very considerable experience and on-the-ground knowledge regarding the challenges that trainees face in completing training.

The main finding of the paper, a year-on-year reduction in trainees graduating from their programmes with fewer operative cases than their peers in previous years, is entirely congruent with the feedback that the SAC has received since training was significantly interrupted by the coronavirus pandemic in 2020 and 2021.

With regards to children’s operative experience, it is true that the curriculum says that trainees should achieve level 4 (“Able to manage without assistance including potential common complications”) for closed reduction of supracondylar fracture of the humerus (SCH), open reduction of SCH, slipped upper femoral epiphysis pinning, anterior drainage of the hip, and operative treatment of femoral shaft fractures (spica, external fixation and flexible nails).^2^ Unfortunately there is a common source of confusion between a level 4 curriculum requirement (as detailed above) and a level 4 procedure-based assessment (PBA). They are not the same thing.

The curriculum mandates that a trainee should be able to manage a particular condition. There is no absolute requirement for the trainee to demonstrate that they can personally and independently perform all of the surgical procedures that can be used to treat that condition. With the increase in network working promoted by the Getting it Right First Time programme and the regional operation delivery networks, it is much more important that a trainee understands their place in the management of these children, some of whom will require treatment in specialist or other centres. The mandatory requirements for paediatric orthopaedics are as follows: to demonstrate operative competency in treatment of SCH by means of a level 4 PBA and completion of case-based discussions in important paediatric orthopaedic conditions that may present on-call. These are currently regarding the painful hip in the child, and the painful spine the child.^3^ It is reassuring to see that the median number of SCH that a trainee has operated on by the end of training is 12. Combined with a level 4 PBA in this procedure it is likely that trainees are competent in the management of this fracture, a common presentation to all hospitals that treat children.

Whether or not a trainee has fulfilled the curriculum requirements is a sophisticated judgement made by an Annual Review of Competence Progression panel based on 6 years of evidence and completed multi-consultant reports in which many consultant colleagues have judged that the trainee has all the skills necessary for the role. We do not find any evidence presented to suggest that trainees have gained their CCT without having appropriate experience in children’s orthopaedics.

Paediatric orthopaedics occupies 11% of training and therefore doing 6 months out of 6 years of training (8.3%) seems appropriate. Some training programmes may be able to deliver these objectives with more focused training, over a shorter period.

We would like to thank the authors for their focus on this important aspect of trauma and orthopaedic training.

## Artificial intelligence

The authors declare that no AI was used to conduct the study or prepare the manuscript.
